# Heterogeneity of fatty acid metabolism in breast cancer cells underlies differential sensitivity to palmitate‐induced apoptosis

**DOI:** 10.1002/1878-0261.12368

**Published:** 2018-08-29

**Authors:** Seher Balaban, Lisa S. Lee, Bianca Varney, Atqiya Aishah, Quanqing Gao, Robert F. Shearer, Darren N. Saunders, Thomas Grewal, Andrew J. Hoy

**Affiliations:** ^1^ Discipline of Physiology Faculty of Medicine and Health School of Medical Sciences & Bosch Institute Charles Perkins Centre The University of Sydney Australia; ^2^ School of Pharmacy Faculty of Medicine and Health The University of Sydney Australia; ^3^ Kinghorn Cancer Center Garvan Institute of Medical Research Darlinghurst Australia; ^4^ School of Medical Sciences UNSW Australia Sydney Australia

**Keywords:** breast cancer, CPT1, DGAT, fatty acid oxidation, oleate, triacylglycerols

## Abstract

Breast cancer (BrCa) metabolism is geared toward biomass synthesis and maintenance of reductive capacity. Changes in glucose and glutamine metabolism in BrCa have been widely reported, yet the contribution of fatty acids (FAs) in BrCa biology remains to be determined. We recently reported that adipocyte coculture alters MCF‐7 and MDA‐MB‐231 cell metabolism and promotes proliferation and migration. Since adipocytes are FA‐rich, and these FAs are transferred to BrCa cells, we sought to elucidate the FA metabolism of BrCa cells and their response to FA‐rich environments. MCF‐7 and MDA‐MB‐231 cells incubated in serum‐containing media supplemented with FAs accumulate extracellular FAs as intracellular triacylglycerols (TAG) in a dose‐dependent manner, with MDA‐MB‐231 cells accumulating more TAG. The differences in TAG levels were a consequence of distinct differences in intracellular partitioning of FAs, and not due to differences in the rate of FA uptake. Specifically, MCF‐7 cells preferentially partition FAs into mitochondrial oxidation, whereas MDA‐MB‐231 cells partition FAs into TAG synthesis. These differences in intracellular FA handling underpin differences in the sensitivity to palmitate‐induced lipotoxicity, with MDA‐MB‐231 cells being highly sensitive, whereas MCF‐7 cells are partially protected. The attenuation of palmitate‐induced lipotoxicity in MCF‐7 cells was reversed by inhibition of FA oxidation. Pretreatment of MDA‐MB‐231 cells with FAs increased TAG synthesis and reduced palmitate‐induced apoptosis. Our results provide novel insight into the potential influences of obesity on BrCa biology, highlighting distinct differences in FA metabolism in MCF‐7 and MDA‐MB‐231 cells and how lipid‐rich environments modulate these effects.

**Abbreviations**
ATF4: activating transcription factor 4
ATGL: adipose triglyceride lipase
BrCa: breast cancer
BSA: bovine serum albumin
CPT1A: carnitine palmitoyltransferase 1A
DGAT‐1: diacylglycerol O‐acyltransferase 1
DMSO: dimethyl sulfoxide
ER: endoplasmic reticulum
FA: fatty acid
FBS: fetal bovine serum
GAPDH: glyceraldehyde 3‐phosphate dehydrogenase
PARP: poly (ADP ribose) polymerase
TAG: triacylglycerols
TLC: thin‐layer chromatography

## 1 Introduction

Cancer cells require adaptations across multiple metabolic processes to support increased rates of cell growth and division (DeBerardinis *et al*., 2008; Hanahan and Weinberg, 2011), highlighting potential new therapeutic strategies. One such adaptive alteration in cancer metabolism observed in most, but not all, cancer cells is termed the ‘Warburg effect’—the upregulation of glycolytic flux and lactate production even in the presence of adequate oxygen (Warburg, 1956). Similarly, demand for glutamine is greater than other nonessential amino acids in cancer cells (Coles and Johnstone, 1962). These alterations are characterized by redistribution of glucose carbons away from catabolism for ATP production toward macromolecule synthesis and maintenance of reductive capacity to sustain proliferation (Romero‐Garcia *et al*., 2011). In addition to enhanced glucose and glutamine metabolism, alterations in cancer cell fatty acid (FA) metabolism have been reported (Balaban *et al*., 2015). However, in general, these observations have predominantly been limited to the synthesis of new FAs from nonlipid carbon sources, that is, glucose and glutamine (Zhang and Du, 2012). One important observation is that FAs contributed more to total ATP turnover in breast cancer (BrCa) cells than that of glucose or glutamine (Guppy *et al*., 2002). However, the influence of readily available FAs in lipid‐rich environments (e.g., from stromal adipocytes or the circulation of obese patients) for utilization in BrCa cells remains to be fully elucidated.

FAs are important metabolic substrates for ATP, NADPH, and macromolecular synthesis and are major regulators in cellular signaling pathways (Glatz and Luiken, 2015). The intracellular‐free FA pool is supplied by *de novo* lipogenesis, intracellular triacylglycerols (TAG) contained in lipid droplets, and exogenous sources—including in the circulation or local microenvironment (Santos and Schulze, 2012). Interestingly, increased lipid droplet number is a feature of aggressive BrCa (Antalis *et al*., 2010; Chamras *et al*., 2002; Przybytkowski *et al*., 2007; Shiu and Paterson, 1984). We recently showed that the vast majority of carbons contributing to the intracellular lipid pool in BrCa cells arise from extracellular FAs and not from glucose or glutamine (Balaban *et al*., 2017). Cells take up FAs from the bloodstream or local microenvironment via various surface transport proteins (Glatz and Luiken, 2017). FAs are then condensed into and stored as TAG in lipid droplets (Farese and Walther, 2009) and other complex lipids including phospholipids (Louie *et al*., 2013), or enter the mitochondria for β‐oxidation (Bruce *et al*., 2009). Interestingly, exogenous FAs have anti‐/pro‐proliferative, promigratory, and antiapoptotic effects in BrCa cells depending on context, suggesting a link between extracellular FAs and BrCa cell behavior (Hardy *et al*., 2000, 2003; Li *et al*., 2014). However, the nature of this effect is dependent on the chemical structure of FAs, the saturation level. Unsaturated FAs, including oleate and linoleate, support cell growth, whereas saturated FAs such as palmitate are toxic to cells (Hardy *et al*., 2000; Listenberger *et al*., 2003). Recently, we showed that coculturing BrCa cells with FA‐rich adipocytes increased TAG levels and promoted BrCa cell proliferation and migration, which were enhanced in the presence of obese adipocytes (Balaban *et al*., 2017). Collectively, these observations suggest a link between intracellular FA metabolism and disease progression that is amplified in obese individuals. However, the mechanistic links between FA availability and metabolism to individual attributes of breast cancer cell behavior are yet to be resolved in detail.

The aim of this study was to characterize the FA metabolism of BrCa cells and their response to FA‐rich environments. Insights into these mechanisms will provide greater understanding of the role that elevated extracellular FA levels may play in linking obesity‐induced enhanced BrCa progression (Eheman *et al*., 2012).

## 2 Materials and methods

### 2.1 Cell culture

The estrogen receptor α‐positive MCF‐7 (ATCC HTB‐22), BT‐474 (ATCC HTB‐20), and MDA‐MB‐175 (ATCC HTB‐25) and the estrogen receptor α‐negative MDA‐MB‐231 (ATCC HTB‐26), BT‐549 (ATCC HTB‐122), and MDA‐MB‐468 (ATCC HTB‐132) human BrCa cells were obtained from the American Type Culture Collection (Manassas, VA, USA) and cultured in high glucose Dulbecco's modified Eagle's medium (DMEM) supplemented with 10% fetal bovine serum (FBS; HyClone, GE Healthcare Life Sciences, Pittsburgh, PA, USA) and 100 IU·mL^−1^ penicillin and 100 IU·mL^−1^ streptomycin (Life Technologies Australia Pty Ltd., Scoresby, Vic, Australia). Cell lines are validated periodically in house by Garvan Molecular Genetics using a test based on the Powerplex 18D Kit (DC1808, Promega, Madison, WI, USA) and tested for mycoplasma every 3 months (MycoAlert™ mycoplasma detection kit, Lonza, Basel, Switzerland).

To inhibit diacylglycerol O‐acyltransferase 1 (DGAT1) activity, cells were treated with AZD3988 (Tocris Bioscience, Invitrogen) (McCoull *et al*., 2012) or dimethyl sulfoxide (DMSO) control for 24 h in low‐glucose DMEM and no antibiotics. After treatment, cells were washed and sensitivity to palmitate‐induced apoptosis was assessed. To inhibit FA oxidation, cells were treated with etomoxir (Sigma‐Aldrich, Castle Hill, NSW, Australia) in low‐glucose DMEM and no antibiotics for times detailed in figure legends.

### 2.2 Lipid‐loading cells

Cells were incubated in medium supplemented with 0–450 μm of either oleate only or 1 : 2 : 1 palmitate/oleate/linoleate (FA mix) as indicated with 10% FBS (vol./vol.) in low‐glucose DMEM and no antibiotics for 24 h.

### 2.3 Substrate metabolism

Extracellular‐derived FA metabolism: Cells were incubated in assay medium containing 0.5 mmol·L^−1^ cold oleate or palmitate, [1‐^14^C]oleate or [1‐^14^C]palmitate (0.5 μCi·mL^−1^; PerkinElmer, Boston, MA), conjugated to 2% (wt/vol.) FA‐free bovine serum albumin (BSA), and 1 mm carnitine in low‐glucose DMEM for 4 h. Mitochondrial oxidation was determined from ^14^CO_2_ production as previously described (Meex *et al*., 2015). Cells were harvested in ice‐cold PBS to determine ^14^C‐oleate incorporation into intracellular lipid pools and protein content.

Cellular lipids were extracted by Folch method (Folch *et al*., 1957). Lipids were separated by thin‐layer chromatography (TLC) using heptane/isopropyl ether/acetic acid (60 : 40 : 3, v/v/v) as developing solvent for TAG or by a two‐step solvent system for ceramides where TLC plates were developed to one‐third of the total length of the plate in chloroform: methanol: 25% NH_3_ (20 : 4 : 0.2, v/v/v), dried, and then re‐chromotographed in heptane/isopropyl ether/acetic acid (60 : 40 : 3, v/v/v). ^14^C activity in the TAG and ceramide bands was determined by scintillation counting.

Intracellular TAG‐derived FA metabolism: Cells were pulsed overnight for 18 h with [1‐^14^C]oleate (1 μCi·mL^−1^; PerkinElmer, Boston, MA, USA) and cold oleate (80 or 450 μm), to prelabel the endogenous TAG pool. Following the pulse, the specific activity of the TAG pool was determined in a cohort of cells by measuring the ^14^C activity in the TAG following lipid extraction and TLC as well as the biochemical assessment of the TAG pool (see Biochemical Measures). Lipolysis was determined in another cohort run in parallel where cells were chased for 4 h in low‐glucose DMEM containing 0.5% FA‐free BSA and 10 μm triacsin C to block FA recycling. TAG‐derived FA oxidation (endogenous FA oxidation) was measured by determination of ^14^CO_2_ production in the absence of triacsin C and in the presence of 1 mm carnitine.

### 2.4 Biochemical measures

Cell TAG were extracted using the method of Folch *et al*. (1957) and quantified using an enzymatic colorimetric method (GPO‐PAP reagent, Roche Diagnostics, North Ryde, NSW, Australia). Cell protein content was determined using Pierce Micro BCA protein assay (Life Technologies Australia Pty Ltd.).

### 2.5 Western blot analysis

Protein extraction was performed as described previously (Hoy *et al*., 2011). Cell lysates were subjected to SDS/PAGE, transferred to PVDF membranes (Merck Millipore, Bayswater, Vic, Australia) and then immunoblotted with antibodies for adipose triglyceride lipase (ATGL), poly (ADP ribose) polymerase (PARP, cleaved PARP), activating transcription factor 4 (ATF4), and glyceraldehyde 3‐phosphate dehydrogenase (GAPDH) obtained from Cell Signaling Technology (Danvers, MA, USA), and anti‐14‐3‐3 from Santa Cruz Biotechnology (ThermoFisher Scientific, Scoresby, Vic, Australia). Chemiluminescent visualization performed using Luminata Crescendo Western Horseradish Peroxidase Substrate (Merck Millipore) and imaged using the Bio‐Rad ChemiDoc MP Imaging System (Bio‐Rad laboratories, Hercules, CA, USA) using image lab software 4.1 (Bio‐Rad laboratories).

### 2.6 Palmitate treatment and cell viability

Cells were plated in triplicate in 96‐well plates (3 × 10^3^ cells/well), and a group of cells were then lipid‐loaded for 24 h. The following day, the media were removed, cells were washed, and fresh low‐glucose DMEM with 10% FBS media ± 250 μm palmitate (Sigma‐Aldrich) added. MTT assays were performed as described previously (Roslan *et al*., 2014) at defined time points stated in figure legends. Viable cells were counted by trypan blue dye exclusion at indicated time points stated in figure legends. In a parallel cohort in 6‐well plates, cells were lysed for immunoblot analysis after 24 h of palmitate treatment.

### 2.7 Gene expression survival analysis

Analysis of DGAT1 gene expression, alteration frequencies, and patient outcomes (overall survival) in all cancers (*n* = 2051) from the METABRIC breast cancer cohort was performed using the cBioPortal for Cancer Genomics (Cerami *et al*., 2012; Gao *et al*., 2013). Hazard ratio was calculated using graphpad prism 7.03 (GraphPad Software, San Diego, CA, USA) from exported primary data from cBioPortal for Cancer Genomics.

### 2.8 Statistical analysis

Statistical analyses were performed with graphpad prism 7.03 (GraphPad Software). Differences among groups were assessed with appropriate statistical tests noted in figure legends. *P *≤* *0.05 was considered significant. Data are reported as mean ± SEM.

## 3 Results

### 3.1 Increasing FA availability increases triacylglycerol content in MCF‐7 and MDA‐MB‐231 cells, leading to increased FA oxidation

**Figure 1:**
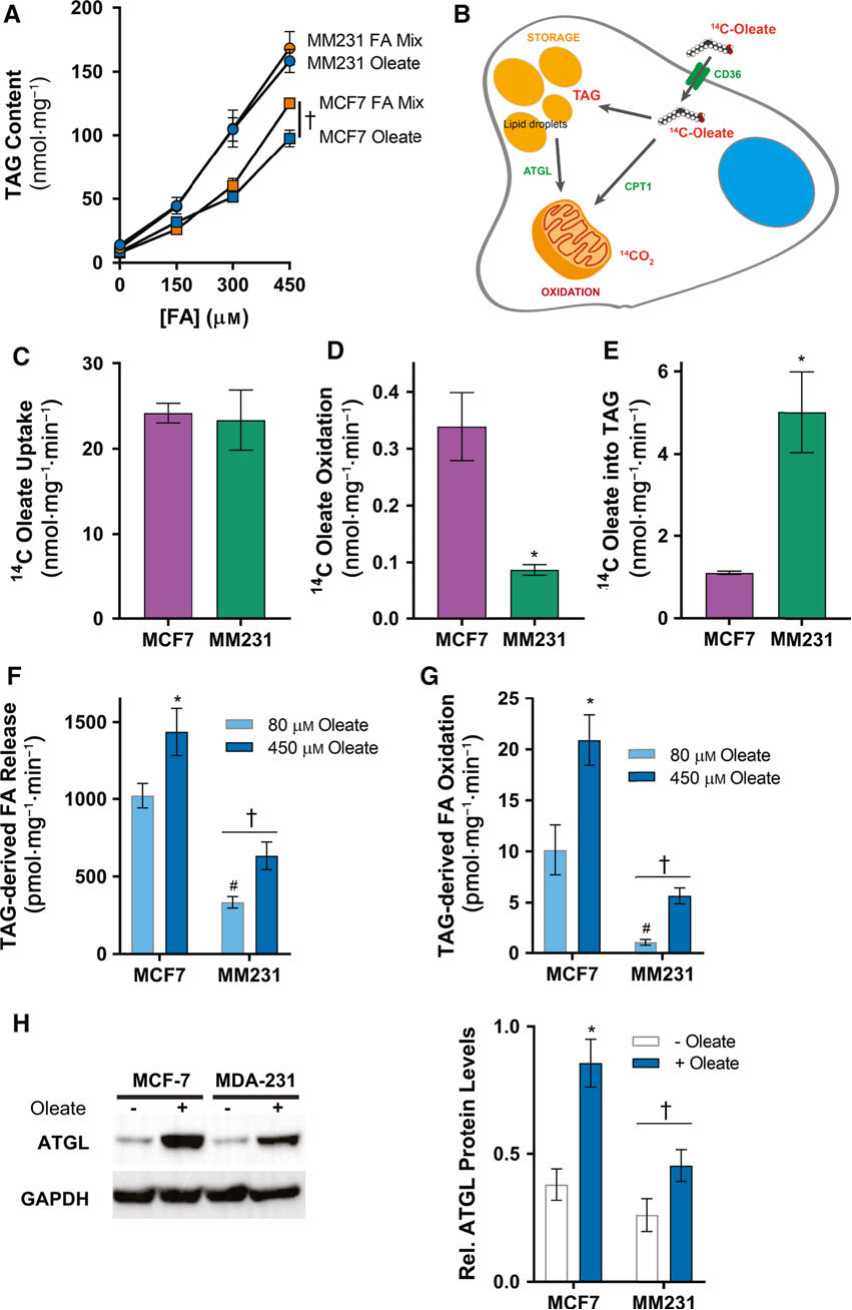
Effect of extracellular lipid availability on intracellular TAG content. (A) MCF‐7 and MDA‐MB‐231 (MM231) cells were treated with a concentration range (0–450 μm) of a lipid cocktail (1 : 2 : 1 palmitate/oleate/linoleate, P/O/L) or oleate alone for 24 h and then assayed for TAG content. (B) Schematic of the pathways mapped by radiotracing of oleate metabolism. ^14^C‐oleate (C) uptake, (D) oxidation, and (E) incorporation into triacylglycerol (TAG) in MCF‐7 and MDA‐MB‐231 cells. *P < 0.05 compared to MCF‐7 by unpaired Student's *t*‐test. (F) MCF‐7 and MDA‐MB‐231 cells were treated with 1 μCi/mL [1‐^14^C]‐oleate and 80 μm or 450 μm cold oleate for 24 h and were subsequently treated with 10 μm triacsin C for 4 h to measure the release of TAG‐derived FAs into lipid‐free media. (G) In another cohort, MCF‐7 and MDA‐MB‐231 cells were incubated with 100 mm carnitine for 4 h and ^14^ CO_2_ production assessed. (H) Representative immunoblots and densitometric quantitation of ATGL in MCF‐7 and MDA‐MB‐231 cells with or without prior overnight incubation with oleate. Data show means ± SEM of three independent experiments performed in triplicate. (A) †*P *≤* *0.05 main effect for cells by three‐way ANOVA. (C–E) *P < 0.05 compared to MCF‐7 by unpaired Student's *t*‐test. (F–H) †*P *≤* *0.05 main effect for cells; *P ≤* *0.05 vs. – oleate; #*P *≤* *0.05 vs. MCF‐7 cells – oleate by two‐way ANOVA followed by Tukey's multiple comparisons test.

We recently reported that adipocyte‐derived FA uptake promotes BrCa cell proliferation (Balaban *et al*., 2017), and others have described that the more aggressive MDA‐MB‐231 cells have increased neutral lipid stores in lipid droplets compared to the less aggressive MCF‐7 cells (Antalis *et al*., 2010; Chamras *et al*., 2002; Przybytkowski *et al*., 2007; Shiu and Paterson, 1984). Firstly, we determined whether the extracellular lipid environment could influence lipid droplet‐contained TAG levels in BrCa cells. MCF‐7 and MDA‐MB‐231 were exposed to a range of oleate concentrations or a FA mix consisting of palmitate, oleate, and linoleate in a 1 : 2 : 1 ratio that represents the mixture of FAs found in the plasma (Watt *et al*., 2012). Total intracellular TAG content of MCF‐7 and MDA‐MB‐231 increased in a dose‐dependent manner in response to increasing concentrations of either oleate alone or the FA mixture (Fig. 1A), showing that the accumulation of FAs in intracellular TAG is not FA species‐specific. Interestingly, MDA‐MB‐231 cells had a higher basal intracellular TAG content (MCF‐7: 8.2 ± 0.6 nmol·mg^−1^; MDA‐MB‐231: 12.7 ± 1.3 nmol·mg^−1^, n = 18; *P *=* *0.004) and a higher capacity to incorporate exogenous FAs into storage compared to MCF‐7 cells (Fig. 1A).

We next examined differences in the capacity of MCF‐7 and MDA‐MB‐231 cells to store extracellular FAs as intracellular TAG (Fig. 1A,B). Interestingly, there was no difference in FA uptake rate (Fig. 1C) but striking differences in intracellular handling of FAs, with MDA‐MB‐231 cells having lower FA oxidation (Fig. 1D) and increased TAG synthesis (Fig. 1E) compared to MCF‐7 cells. The differences in FA oxidation align with our previously reported observations of elevated carnitine palmitoyltransferase 1A (CPT1A) protein levels in MCF‐7 cells compared to MDA‐MB‐231 cells, with MCF‐7 cells have greater amounts of CPT1A compared to MDA‐MB‐231 cells (Balaban *et al*., 2017).

Lipid droplet‐contained TAG is a temporary storage destination for FAs and acts as an easily mobilized supply of intracellular FAs, so we investigated whether increased intracellular lipid pools altered FA metabolism in these cells. Overnight exposure to media containing an additional 450 μm oleate and 0.2 μCi·mL^−1^ ^14^C‐oleate increased TAG levels in both MCF‐7 and MDA‐MB‐231 cells compared to cells treated with 80 μm oleate and 0.2 μCi·mL^−1^ ^14^C‐oleate (data not shown, similar to Fig. 1A). The release of FA into the media (i.e., lipolysis) was significantly greater from MCF‐7 cells compared to MDA‐MB‐231 cells (Fig. 1F). Further, overnight lipid‐loading increased lipolysis in both cell lines (Fig. 1F). Similar patterns were observed in the oxidation of intracellular TAG‐derived FAs (Fig. 1G), which was consistent with differences in the protein levels of the TAG lipase ATGL (Fig. 1H). Therefore, these experiments demonstrate that 1) MDA‐MB‐231 cells accumulate more FAs as TAG due to low oxidative and TAG hydrolysis rates and increased flux of FAs into TAG and 2) MCF‐7 cells have high oxidative and TAG hydrolysis rates and low rate of FA incorporation into TAG.

### 3.2 MCF‐7 cells have mild sensitivity to palmitate‐induced apoptosis

**Figure 2:**
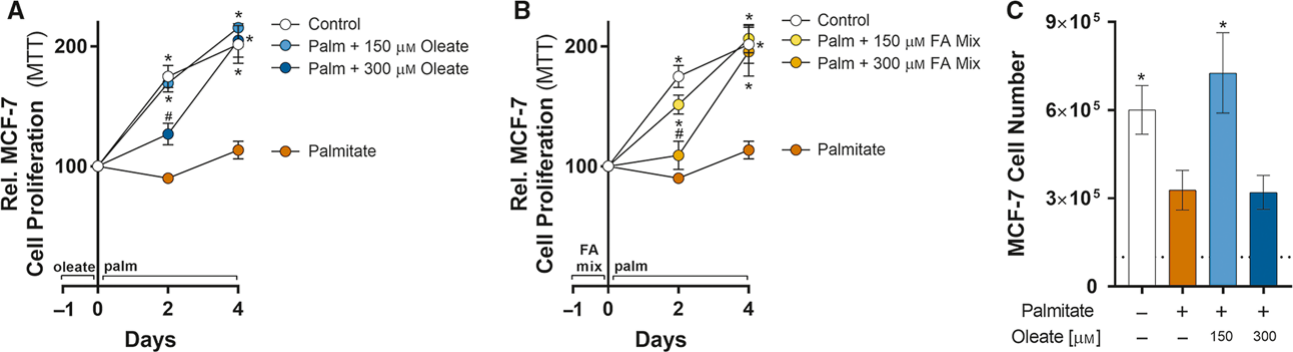
The MCF‐7 cells are not sensitive to palmitate‐induced apoptosis. MTT assays of MCF‐7 cells treated with 250 μm palmitate for 4 days with or without prior overnight incubation with (A) oleate or (B) 1 : 2 : 1 mixture of palmitate/oleate/linoleate (FA mix). MTT results are presented as percentages of MTT absorbance at indicated time points relative to that at Day 0 for each group (five independent experiments performed in quadruplicate). (C) Cell number of MCF‐7 cells treated with 250 μm palmitate for 4 days with or without prior overnight incubation with oleate. The dashed line represents the number of cells present at Day 0 (three independent experiments performed in triplicate). Data are presented as mean ± SEM. *P ≤* *0.05 vs. palmitate; #*P *≤* *0.05 vs. control by two‐way ANOVA (A and B) or one‐way ANOVA (D) followed by Tukey's multiple comparisons test.

Both MDA‐MB‐231 and MCF‐7 cells have the capacity to accumulate TAG following incubation in FA‐rich media (Fig. 1). Although FA accumulation in lipid droplets of BrCa cells is well‐described (Antalis *et al*., 2010; Chamras *et al*., 2002; Przybytkowski *et al*., 2007; Shiu and Paterson, 1984), the role of this lipid‐rich, energy‐dense metabolic substrate in cellular behavior is not well defined. While exogenous FAs have both anti‐ and pro‐proliferative effects as well as promigratory and antiapoptotic effects in BrCa cells (Hardy *et al*., 2000, 2003; Louie *et al*., 2013), little is known about the responsiveness of lipid‐loaded BrCa cells to apoptotic stimuli. To assess this, we challenged MCF‐7 cells to high levels of palmitate in serum‐containing media, which can induce apoptosis (Hardy *et al*., 2000, 2003; Kamili *et al*., 2015; Listenberger *et al*., 2003). MCF‐7 cells incubated in palmitate‐containing media displayed a striking attenuation in cell viability, as judged by MTT signal (Fig. 2A,B) and cell number (Fig. 2C)—suggesting either inhibition of cell proliferation and/or induction of apoptosis—compared to cells grown in FBS only media. We failed to detect evidence that apoptotic signaling was activated by the addition of palmitate to serum‐containing media (data not shown). Lipid‐loaded MCF‐7 cells with either oleate alone (Fig. 2A) or FA mix (Fig. 2B) were protected from this palmitate‐induced reduction in MTT and cell number (Fig. 2C). These results suggest that extracellular FA availability, and the associated increase in intracellular lipid stores, is associated with protection of MCF‐7 cells from palmitate‐induced apoptosis.

### 3.3 Increasing intracellular TAG levels protects MDA‐MB‐231 cells from palmitate‐induced apoptosis and serum starvation

Palmitate treatment reduced cell proliferation in MCF‐7 cells, and exposing these cells to additional extracellular FAs protected cells from palmitate‐induced apoptosis. The same approach was used in MDA‐MB‐231 cells in order to determine whether BrCa cell lines of different hormone receptor status show varied response to palmitate.

**Figure 3:**
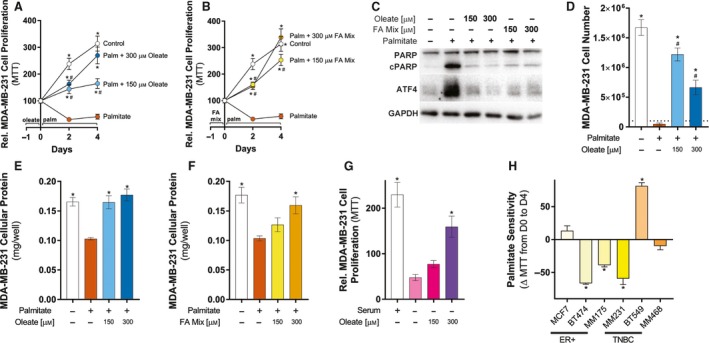
The MDA‐MB‐231 cells are sensitive to palmitate‐induced apoptosis and lipid‐loading protects MDA‐MB‐231 cells from palmitate‐induced apoptosis. MTT assays of MDA‐MB‐231 cells treated with 250 μm palmitate for 4 days with or without prior overnight incubation with (A) oleate or (B) 1 : 2 : 1 mixture of palmitate/oleate/linoleate (FA mix). MTT results are presented as percentages of MTT absorbance at indicated time points relative to that at Day 0 for each group. (five independent experiments performed in quadruplicate). (C) Representative immunoblots of cPARP and ATF4 levels of MDA‐MB‐231 cells treated with 250 μm palmitate for 1 day with or without prior overnight incubation with oleate or 1 : 2 : 1 mixture of palmitate/oleate/linoleate (FA mix; representative of three independent experiments performed in triplicate). (D) Cell number of MDA‐MB‐231 cells treated with 250 μm palmitate for 4 days with or without prior overnight incubation with oleate. The dashed line represents the number of cells present at Day 0 (three independent experiments performed in duplicate). Cell protein content of MDA‐MB‐231 cells treated with 250 μm palmitate for 4 days with or without prior overnight incubation with (E) oleate or (F) 1 : 2 : 1 mixture of palmitate/oleate/linoleate (FA mix) (three independent experiments performed in duplicate). (G) MTT assays of MDA‐MB‐231 cells serum‐starved for 4 days with or without prior overnight incubation with oleate. (H) Palmitate sensitivity of MCF‐7, BT‐474, MDA‐MB‐175, MDA‐MB‐231, BT‐549, and MDA‐MB‐468 cells expressed as the difference in MTT signal between Day 0 (D0) and Day 4 (D4) for cells cultured in serum‐containing media supplemented with 250 μm. Data are presented as mean ± SEM. *P ≤* *0.05 vs. palmitate; #*P *≤* *0.05 vs. control by two‐way ANOVA (A and B) or one‐way ANOVA (D–F) followed by Tukey's multiple comparisons test. *P ≤* *0.05 vs. Day 0 MTT signal by Student's *t*‐test (H).

The addition of 250 μm palmitate to FBS media strikingly reduced MTT absorbance within 2 days, reducing even further by 4 days compared to cells cultured in FBS (Fig. 3A,B). This reduced reductive metabolic capacity (measured via MTT assay) was associated with activation of PARP and ATF4 signaling after 1 day of palmitate treatment (Fig. 3C), which are markers of apoptosis (Elmore, 2007; Sano and Reed, 2013). Further, the early activation of apoptotic signaling corresponded with reduced cell number (Fig. 3D) and cellular protein amount (Fig. 3E,F) following 4 days of palmitate treatment. Similar to MCF‐7 cells, MDA‐MB‐231 cells lipid‐loaded with either oleate alone (Fig. 3A) or FA mixture (Fig. 3B) were partly protected from the effects of palmitate‐induced reduction in MTT activity. Further, the effect of palmitate on PARP and ATF4 signaling (Fig. 3B), cell number (Fig. 3D), and cellular protein amount (Fig. 3E,F) was reduced by prior incubation of either oleate alone or FA mixture. In the absence of serum, lipid‐loaded MDA‐MB‐231 cells were similarly, but less strikingly, protected from serum starvation‐induced apoptosis (Fig. 3G). Collectively, these data demonstrate that MDA‐MB‐231 cells are highly sensitive to palmitate‐induced apoptosis, whereas MCF‐7 have attenuated cell proliferation when cultured in palmitate‐rich media, and that intracellular lipid stores influence MCF‐7 and MDA‐MB‐231 sensitivity to palmitate‐induced apoptosis.

To complement our novel observations of heterogeneity in the response of MCF‐7 and MDA‐MB‐231 cells to palmitate supplementation, we assessed the sensitivity of other BrCa cells to palmitate and whether this effect was altered by prior lipid‐loading. Unlike MCF‐7 cells, BT‐474 and MDA‐MB‐175 cells incubated in palmitate‐containing media displayed a striking reduction in MTT signal (Fig. S1A–D) and cells lipid‐loaded with either oleate alone (Fig. S1A and C) or FA mixture (Fig. S1B and D) were partly protected from the effects of palmitate. Interestingly, BT‐549 cells cultured in palmitate‐containing media displayed attenuated cell growth (Fig. S1E and F), whereas MDA‐MB‐468 cells more closely phenocopied MDA‐MB‐231 cells in that palmitate supplementation of serum‐containing media induced apoptosis which was partly prevented by prior oleate (Fig. S1G) or FA mixture (Fig. S1H) treatment. Overall, we observed no consistent response to palmitate supplementation across the three estrogen receptor α‐positive (MCF‐7, BT‐474, and MDA‐MB‐175), and three estrogen receptor α‐negative (MDA‐MB‐231, BT‐549, and MDA‐MB‐468) human BrCa cells (Fig. 3H).

### 3.4 Differences in palmitate handling explain the differential response to palmitate‐induced apoptosis in MCF‐7 and MDA‐MB‐231 cells

**Figure 4:**
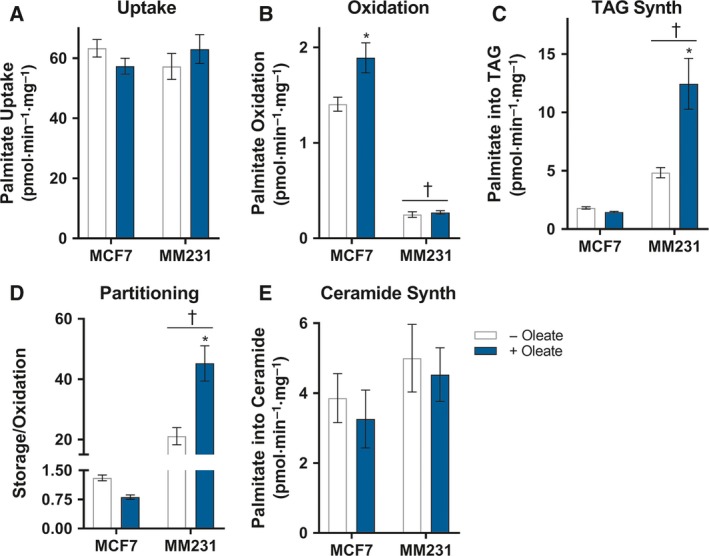
The MCF‐7 and MDA‐MB‐231 cells metabolize palmitate differently and this is selectively altered by pretreatment with oleate. ^14^C‐palmitate (A) uptake, (B) oxidation, (C) incorporation into triacylglycerol (TAG), (D) intracellular partitioning of ^14^C‐palmitate expressed as the ratio of ^14^C‐palmitate incorporation into triacylglycerol (storage) vs. ^14^C‐palmitate oxidation, ^14^C‐palmitate incorporation into (E) ceramide in MCF‐7 and MDA‐MB‐231 cells with or without prior overnight incubation with 150 μm oleate. Data are presented as mean ± SEM of three independent experiments performed in triplicate. †*P *≤* *0.05 main effect for cells; *P ≤* *0.05 vs. – oleate; #*P *≤* *0.05 vs. MCF‐7 cells – oleate by two‐way ANOVA followed by Tukey's multiple comparisons test.

The striking differences in palmitate sensitivity between MDA‐MB‐231 and MCF‐7 cells, both in the basal state and after lipid‐loading, may be due to altered intracellular palmitate metabolism, similar to the patterns of oleate metabolism (Fig. 1). To test this, the fate of extracellularly‐derived radiolabeled FAs was assessed in MDA‐MB‐231 and MCF‐7 cells incubated in ^14^C‐palmitate, in either the basal state or following overnight exposure to oleate. Total palmitate uptake was similar in MDA‐MB 231 and MCF‐7 cells, and we observed no effect of overnight oleate exposure on palmitate uptake (Fig. 4A). Interestingly, MDA‐MB‐231 cells had dramatically lower palmitate oxidation (Fig. 4B) and greater incorporation of palmitate into TAG (Fig. 4C) compared to MCF‐7 cells, consistent with our previous observations of oleate metabolism (Balaban *et al*., 2017). Pretreatment with oleate increased palmitate oxidation in MCF‐7 but not MDA‐MB‐231 cells (Fig. 4B), whereas MDA‐MB‐231 TAG synthesis was increased (Fig. 4C). Hence, the overall effect is a greater partitioning of palmitate into storage relative to oxidation in MDA‐MB‐231 cells compared to MCF‐7 cells, and this intracellular partitioning in MDA‐MB‐231 cells is increased by pretreatment with oleate (Fig. 4D).

Palmitate is a critical precursor of *de novo* ceramide synthesis (Kitatani *et al*., 2008), which can activate apoptosis (Tohyama *et al*., 1999). As such, one hypothesis to explain the enhanced sensitivity to palmitate in MDA‐MB‐231 cells compared to MCF‐7 cells was enhanced ceramide synthesis in MDA‐MB‐231 cells. However, there was no difference in the rate of palmitate incorporation into ceramide in MCF‐7 and MDA‐MB‐231 cells, and this was not altered by oleate pretreatment (Fig. 4E), thereby excluding this mechanism. Collectively, these experiments demonstrate that MCF‐7 and MDA‐MB‐231 cells incorporate exogenous palmitate at similar rates, but they metabolize this saturated FA differently. Specifically, MCF‐7 cells have higher rates of palmitate oxidation compared to MDA‐MB‐231 cells, whereas MDA‐MB‐231 cells have a higher rate of storing palmitate as TAG and this is enhanced by pretreatment with oleate. As such, the differences in palmitate handling may explain the differential sensitivity to palmitate‐induced apoptosis.

### 3.5 Inhibition of mitochondrial FA oxidation sensitizes MCF‐7 cells to palmitate‐induced apoptosis

**Figure 5:**
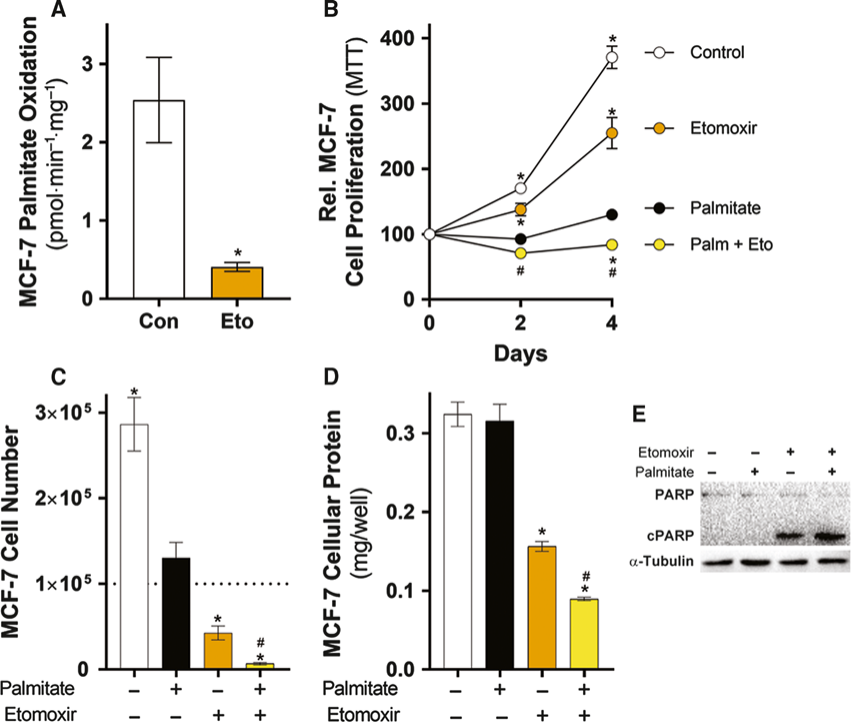
Inhibition of fatty acid oxidation in MCF‐7 cells sensitizes cells to palmitate‐induced apoptosis. (A) ^14^C‐palmitate oxidation in MCF‐7 cells that were treated with or without 100 μM etomoxir (Eto) (five independent experiments performed in triplicate). (B) MTT assays of MCF‐7 cells treated with 250 μm palmitate (Palm), 100 μm etomoxir (Eto), or a combination for 4 days. MTT results are presented as percentages of MTT absorbance at indicated time points relative to that at Day 0 for each group (MTT: six independent experiments performed in quadruplicate). (C) Cell number and (D) protein amount of MCF‐7 cells treated with 250 μm palmitate (Palm), 100 μm etomoxir (Eto), or a combination for 4 days. The dashed line represents the number of cells present at Day 0 (three independent experiments performed in triplicate). (E) Representative immunoblots of cPARP of MCF‐7 cells treated with 250 μm palmitate, 100 μm etomoxir, or a combination for 1 day (three independent experiments performed in triplicate). Data are presented as mean ± SEM. (A) *P ≤* *0.05 vs. control by unpaired Student's t‐test. (B) *P ≤* *0.05 vs. palmitate; #*P *≤* *0.05 vs. etomoxir by two‐way ANOVA followed by Tukey's multiple comparisons test. (C and D) *P ≤* *0.05 vs. palmitate; #*P *≤* *0.05 vs. etomoxir by one‐way ANOVA followed by Tukey's multiple comparisons test.

MCF‐7 cells are protected from palmitate‐induced apoptosis compared to MDA‐MB‐231 cells, which may be due to higher palmitate oxidation (Fig. 4B) related to CPT1A protein levels (Balaban *et al*., 2017). Therefore, we tested whether inhibiting palmitate oxidation sensitized MCF‐7 cells to palmitate‐induced apoptosis. Treating MCF‐7 cells with the CPT1 inhibitor etomoxir lowered basal palmitate oxidation (Fig. 5A). The addition of 250 μm palmitate to growth media slowed cell growth but the combination of palmitate and etomoxir further reduced the MTT signal (Fig. 5B). This reduction in MTT signal was associated with reduced MCF‐7 cell number (Fig. 5C) and cellular protein amount (Fig. 5D) after 4 days of treatment, as well as activation of PARP signaling after 1 day of treatment (Fig. 5E). Inhibition of FA oxidation sensitizes MCF‐7 cells to palmitate‐induced apoptosis, indicating that FAO is an important part of apoptosis resistance in these cells. There were some discrepancies in the measured effect of palmitate and etomoxir alone between individual readouts (i.e., MTT, cell number, and cellular protein), which likely reflect differential effects on the cellular characteristic being measured. For example, MTT is a redox/cell viability measure which may not necessarily correlate with cell number and cellular protein levels in all instances.

### 3.6 Inhibition of oleate‐stimulated TAG synthesis restores sensitivity to palmitate‐induced apoptosis in lipid‐loaded MDA‐MB‐231 cells

**Figure 6:**
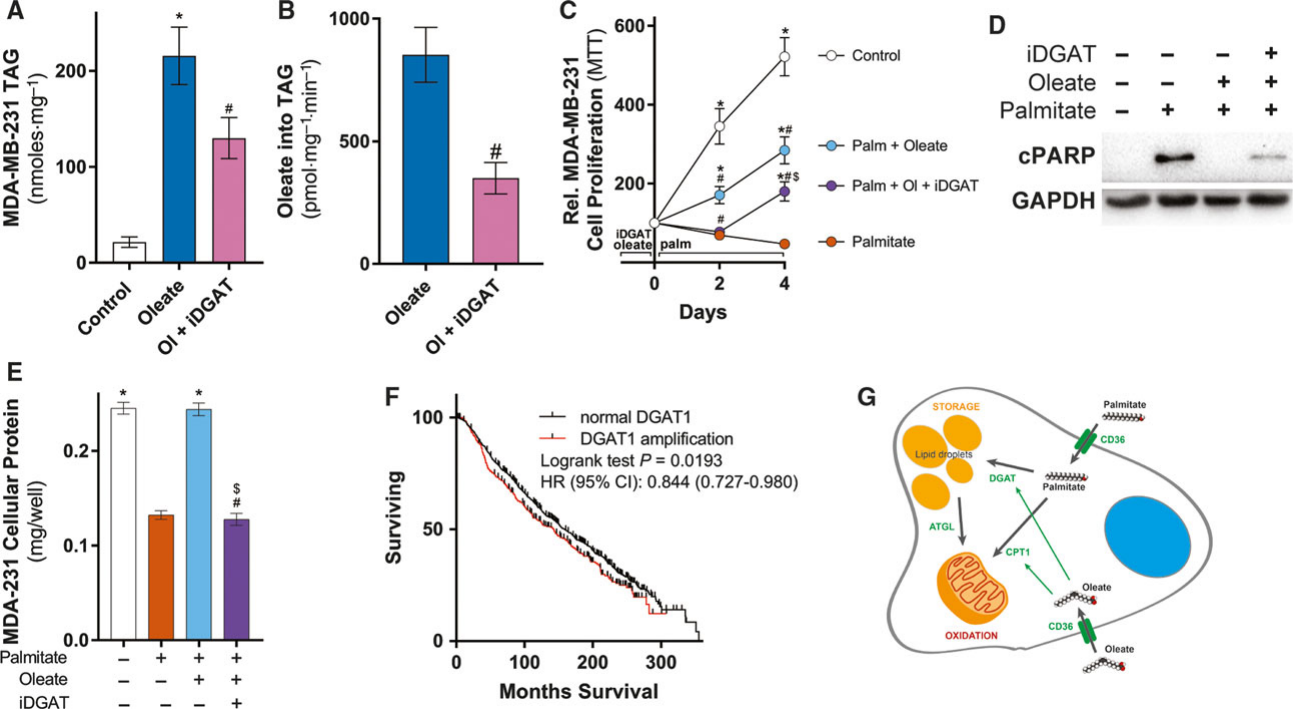
Inhibition of TAG synthesis in MDA‐MB‐231 cells blunts the protective effects of prior oleate treatment to palmitate‐induced apoptosis. (A) MDA‐MB‐231 cell triacylglycerol (TAG) levels in cells treated with 300 μm oleate with or without 0.6 nm DGAT inhibitor AZD3988 (iDGAT) for 24 h (four independent experiments performed in duplicate). (B) ^14^C‐oleate incorporation into TAG in MDA‐MB‐231 cells that were treated with or without DGAT inhibitor (iDGAT; three independent experiments performed in duplicate). (C) MTT assays of MDA‐MB‐231 cells treated with 250 μm palmitate (Palm) for 4 days following prior incubation with 300 μM oleate (Ol) with or without 0.6 nm DGAT inhibitor AZD3988 (iDGAT). MTT results are presented as percentages of MTT absorbance at indicated time points relative to that at Day 0 for each group (four independent experiments performed in quadruplicate). (D) Representative immunoblots of cPARP of MDA‐MB‐231 cells treated with 250 μm palmitate for 1 days following prior incubation with oleate with or without DGAT1 inhibitor (three independent experiments performed in triplicate). (E) Protein amount of MDA‐MB‐231 cells treated with 250 μm palmitate for 4 days following prior incubation with 300 μM oleate with or without DGAT inhibitor (iDGAT; three independent experiments performed in triplicate). (F) Differential overall survival among breast cancer cases parsed by DGAT1 amplification. (G) Schematic of the mechanisms by which oleate pretreatment prevents palmitate‐induced apoptosis. Data are presented as mean ± SEM. (A) *P ≤* *0.05 vs. control; #*P *≤* *0.05 vs. oleate by one‐way ANOVA followed by Tukey's multiple comparisons test. (B) #*P *≤* *0.05 vs. oleate by unpaired Student's *t*‐test. (C) *P ≤* *0.05 vs. palmitate; #*P *≤* *0.05 vs. control; $*P *≤* *0.05 vs. palm + oleate by two‐way ANOVA followed by Tukey's multiple comparisons test. (E) *P ≤* *0.05 vs. palmitate; #*P *≤* *0.05 vs. control; $*P *≤* *0.05 vs. palm + oleate by one‐way ANOVA followed by Tukey's multiple comparisons test.

Pretreating MDA‐MB‐231 cells with either oleate alone or the FA mixture protected from apoptosis induced by either palmitate or serum starvation (Fig. 3). Radiometric analysis of palmitate metabolism points to an increase in TAG synthesis (Fig. 4C) as a potential mechanism by which FA pretreatment protects from palmitate apoptosis by shunting palmitate into lipid droplet‐contained TAG. We directly tested this by inhibiting TAG synthesis through the addition of an inhibitor of DGAT1, which catalyzes the final reaction in TAG synthesis (Cases *et al*., 1998, 2001), only during the preincubation of oleate prior to palmitate treatment. As expected, DGAT1 inhibition attenuated the increase in MDA‐MB‐231 TAG content in response to oleate administration (Fig. 6A) as a consequence of a reduction in the rate of incorporation of radiolabeled oleate into TAG (Fig. 6B). As previously observed, palmitate treatment induced apoptosis and preincubation with oleate blunted this effect (Fig. 6C); however, lowering intracellular TAG levels by DGAT1 inhibition in the presence of oleate pretreatment restored MDA‐MB‐231 cell sensitivity to palmitate at Day 2 (Fig. 6C). After 4 days of palmitate treatment, the MTT signal in MDA‐MB‐231 cells pretreated with the DGAT1 inhibitor and oleate did increase but it was still lower than MDA‐MB‐231 cells that were pretreated with oleate alone (Fig. 6C). The reduction in MTT signal was associated with reactivation of PARP signaling (Fig. 6D) and reduced cellular protein amount (Fig. 6E). Therefore, TAG synthesis is required for the protective effects of pretreating MDA‐MB‐231 cells with FAs to palmitate‐induced apoptosis. Analysis of the METABRIC breast cancer cohort (Clark, 1989; Curtis *et al*., 2012) showed DGAT1 amplification in 21% (434/2051) of breast cancer samples. Analysis of overall survival in this cohort showed a significant decrease in overall survival in patients with DGAT1 amplification (143 months vs 158.6 months, HR: 0.844 (95% CI: 0.727–0.980), *P *=* *0.0193; Fig. 6F).

## 4 Discussion

Cancer cells require adaptations across multiple metabolic processes to support increased rate of cell growth and division (DeBerardinis *et al*., 2008). The majority of interest in tumor metabolism to date has focused on glucose metabolism (e.g., Warburg effect) and glutamine addiction in cancer cells (Ward and Thompson, 2012). FAs have received far less attention, even though the contribution of FAs to total ATP turnover in BrCa cells is greater than that of glucose or glutamine (Guppy *et al*., 2002). In this study, we demonstrate that MCF‐7 and MDA‐MB‐231 cells differ in their intracellular handling of FAs and in response to palmitate‐induced apoptosis. These differences in palmitate‐induced apoptosis were reflected across a range of other BrCa cell lines, independent of estrogen receptor α‐status, highlighting the striking heterogeneity in palmitate sensitivity. Specifically, MCF‐7 cells have increased FA oxidative capacity that underpins their resistance to palmitate‐induced apoptosis. MDA‐MB‐231 cells have increased TAG synthesis capacity and are highly sensitive to palmitate‐induced apoptosis which was inhibited by prior lipid‐loading to stimulate TAG synthesis.

All cells and organisms store lipids to provide a buffer for energy fluctuations and promote survival. This lipid is stored in cytosolic lipid droplets, which are often in close proximity to other cellular organelles, notably mitochondria and endoplasmic reticulum (ER). Lipid droplets are highly conserved in yeast through to mammals (Le Lay and Dugail, 2009), but the size and number of lipid droplets varies between cell types. Lipid droplets store neutral lipids including TAG and cholesteryl esters as temporary complex lipid storage forms of FAs and cholesterol. A number of studies have reported that the number of lipid droplets in estrogen receptor α‐positive MCF‐7 cells is lower than in the more aggressive ERα‐negative MDA‐MB‐231 cells (Antalis *et al*., 2010; Chamras *et al*., 2002; Przybytkowski *et al*., 2007; Shiu and Paterson, 1984). Consistent with these observations, we demonstrated that both MCF‐7 and MDA‐MB‐231 cells can respond to changing levels of extracellular FAs and accumulate these as TAG in a dose‐dependent manner, independent of FA species. Interestingly, MDA‐MB‐231 cells accumulated more FAs in TAG compared to MCF‐7 cells, which is supported by a previous report that investigated the addition of oleate only to culture media (Przybytkowski *et al*., 2007). This increased ability of MDA‐MB‐231 cells to accumulate more TAG is a consequence of increased partitioning of FAs into TAG, and lower oxidative and TAG lipolytic capacity compared to MCF‐7 cells. The lower rates of FA oxidation and lipolysis correlate with protein levels of the rate limiting enzymes in these pathways, CPT1 (Balaban *et al*., 2017) and ATGL, respectively. These observations suggest that MDA‐MB‐231 cells are more sensitive to extracellular FA availability and have higher capacity to store FAs as TAG than MCF‐7 cells. However, the role of estrogen receptor α‐mediated signaling in driving the differences in FA metabolism between these two cell lines remains to be determined.

Exposure to elevated levels of the saturated FA palmitate induces apoptosis in a range of cells including 3T3 fibroblasts (Kamili *et al*., 2015), peripheral blood mononuclear cells (RostamiRad *et al*., 2018), human cardiac progenitor cells (Leonardini *et al*., 2017), pancreatic β cells (Boslem *et al*., 2011; Luo *et al*., 2017), macrophages (Kim *et al*., 2017), and hepatocytes (Penke *et al*., 2017). Palmitate also impairs MDA‐MB‐231 cell proliferation and activates apoptosis (Baumann *et al*., 2016; Hardy *et al*., 2000, 2003; Kourtidis *et al*., 2009; Wu *et al*., 2017) via a number of mechanisms, including ER stress (Baumann *et al*., 2016; Boslem *et al*., 2011), impaired autophagy (RostamiRad *et al*., 2018; Wu *et al*., 2017), altered NAD metabolism (Penke *et al*., 2017), and ceramide synthesis (Luo *et al*., 2017). Importantly, many of the deleterious effects of palmitate on cellular function are mitigated by cotreatment with other FAs, in particular the monounsaturated FA oleate (Colvin *et al*., 2017; Kim *et al*., 2017; Penke *et al*., 2017; Sargsyan *et al*., 2016), which itself is pro‐proliferative and activates phosphoinositide 3‐kinase signaling (Hardy *et al*., 2000).

We provide new insight into both the cell‐specific effects of palmitate as well as the mechanism by which palmitate alters BrCa cell biology. Firstly, MCF‐7 cells are less sensitive to the apoptotic effects of palmitate treatment in the presence of serum compared to MDA‐MB‐231 cells, which is similar in response to serum starvation (Przybytkowski *et al*., 2007), and palmitate treatment in serum‐free conditions (Hardy *et al*., 2000). The differential response was not due to differences in palmitate uptake but associated with postuptake intracellular handling of palmitate. Specifically, MDA‐MB‐231 cells partition palmitate toward storage as lipid droplet confined TAG, likely explaining the differences in lipid droplet amount between MDA‐MB‐231 and MCF‐7 cells (Antalis *et al*., 2010; Chamras *et al*., 2002; Przybytkowski *et al*., 2007; Shiu and Paterson, 1984). MCF‐7 cells preferentially partition palmitate toward mitochondrial oxidation, which is associated with increased CPT1A protein levels in these cells (Balaban *et al*., 2017). Mitochondrial FA oxidation is the primary bioenergetic pathway in many nontumor tissues (Bonnefont *et al*., 2004) and is a greater contributor to total ATP turnover than glucose or glutamine in MCF‐7 cells (Guppy *et al*., 2002). Pharmacological inhibition of this metabolic pathway impairs cell growth and viability in a range of cancer cells (Rodriguez‐Enriquez *et al*., 2015), including MYC‐driven triple‐negative BrCa (Camarda *et al*., 2016). We and others have reported that MCF‐7 cells have high rates of mitochondrial oxidation of exogenous FAs compared to MDA‐MB‐231 cells (Przybytkowski *et al*., 2007), and we now report that this is also the case for the oxidation of intracellular‐derived FAs. This elevated FA oxidative capacity in MCF‐7 cells was associated with higher levels of CPT1A (Balaban *et al*., 2017) and ATGL protein levels, and was enhanced by overnight oleate treatment. We have recently reported that CPT1A and oxidative phosphorylation protein levels and FA oxidation rate are also increased following coculture with adipocytes (Balaban *et al*., 2017), which was also observed with ZR‐75‐1 BrCa cells (Wang *et al*., 2017). In fact, inhibition of FA oxidation reduced adipocyte‐stimulated ZR‐75‐1 BrCa cell invasion (Wang *et al*., 2017) and we report here that inhibition of FA oxidation sensitized MCF‐7 cells to palmitate‐induced apoptosis, as determined by a combination of MTT, cell number, cellular protein amount, and PARP signaling. Our results shed new light on the contribution of FA oxidation in MCF‐7 cells where pharmacological inhibition of FA oxidation sensitized MCF‐7 cells to palmitate‐induced apoptosis and suggests that targeting FA oxidation is an attractive therapeutic strategy in BrCa. The addition or presence of oleate ameliorates the cytotoxic effects of palmitate (Capel *et al*., 2016; Colvin *et al*., 2017; Kim *et al*., 2017; Kwon and Querfurth, 2015; Penke *et al*., 2017; Sargsyan *et al*., 2016). Proposed mechanisms for these observations include attenuating palmitate‐induced ER stress (Colvin *et al*., 2017), preventing activation of the unfolded protein response (Sommerweiss *et al*., 2013), activating prosurvival pathways of ER stress (Sargsyan *et al*., 2016), restoring insulin stimulated protein kinase B (Akt) signaling (Capel *et al*., 2016), and activating AMP‐activated protein kinase and mechanistic target of rapamycin signaling (Kwon and Querfurth, 2015). Oleate treatment in a range of cells also modulates palmitate metabolism including stimulating TAG synthesis, preventing diacylglycerol accumulation (Capel *et al*., 2016; Kwon *et al*., 2014), and preventing mitochondrial reactive oxygen species production and dysfunction (Kwon *et al*., 2014). These observations were predominantly made by cotreatment with oleate and palmitate. In the current study, we also observed that pretreatment with either oleate or a FA mixture to increase intracellular TAG levels prevented palmitate‐induced apoptosis. This alteration in the response to palmitate treatment was observed in both MCF‐7 and MDA‐MB‐231 cells and was associated with increased palmitate oxidation in MCF‐7 cells and increased TAG synthesis in MDA‐MB‐231 cells. There was no change in palmitate uptake or palmitate incorporation into ceramide following overnight oleate treatment. This is counter to the recent report that oleate cotreatment of RAW264.7 macrophage cells prevented the palmitate‐induced increase in the mRNA level of the FA transporter CD36 (Kim *et al*., 2017), hinting at cell type‐specific mechanisms. Collectively, oleate and FA treatment influences MCF‐7 and MDA‐MB‐231 cell palmitate metabolism and thereby avoid the deleterious effects of lipotoxicity.

Several studies have demonstrated that the ability to synthesize TAG plays a critical role in the protection from palmitate‐induced lipotoxicity (Kamili *et al*., 2015; Listenberger *et al*., 2003; Przybytkowski *et al*., 2007). For example, oleate supplementation promotes TAG synthesis in CHO cells and also prevents palmitate‐induced apoptosis in mouse embryonic fibroblasts but this protection was not seen in DGAT1^−/−^ mouse embryonic fibroblasts (Listenberger *et al*., 2003). We demonstrate that pretreatment of MDA‐MB‐231 cells with oleate or FA mix protects from both palmitate‐induced and serum starvation‐induced apoptosis. The ability of oleate pretreatment to prevent serum starvation‐induced apoptosis is consistent with previous observations in other models (Przybytkowski *et al*., 2007). The pro‐apoptotic effects of palmitate in MDA‐MB‐231 cells has been shown to require autophagy protein 5 (Wu *et al*., 2017), whereas we show that the protection from palmitate‐induced apoptosis by prior lipid‐loading required the upregulation of TAG synthesis via DGAT1. Further, DGAT1 amplification in a large breast cancer cohort was associated with a significant decrease in overall survival. This latter observation was not seen in H4IIEC3 rat hepatoma cells when inhibiting TAG synthesis through siRNA silencing of DGAT1 and DGAT2 (Leamy *et al*., 2016). Although MDA‐MB‐231 cells have a high incorporation rate of FAs into intracellular TAG pools compared to MCF‐7 cells, this rate appears to be inadequate to prevent palmitate‐induced apoptosis and only with cosupplementation with oleate or pretreatment with oleate or the FA mix is the synthesis of TAG sufficient to partition lipotoxic palmitate into safe storage in intracellular lipid droplet‐contained TAG.

This study has focused on the contribution of FA metabolism in mediating palmitate‐induced apoptosis. Extensive literature exists describing the effects of FAs on BrCa cell proliferation and migration/invasion (see review (Kinlaw *et al*., 2016)). For example, oleate increases proliferation, migration and invasion in MDA‐MB‐231 cells (Hardy *et al*., 2005; Wu *et al*., 2017) and MCF‐7 cells (Soto‐Guzman *et al*., 2008), but this effect on MCF‐7 cells is not always observed (Hardy *et al*., 2005). Other literature has described the effects of other FA species such as the omega‐3 or omega‐6 FAs on BrCa cell biology (Mansara *et al*., 2015; Tiwari *et al*., 1991; Zhang *et al*., 2012). However, the breadth of FA diversity, driven by differences in chain length and saturation/desaturation status, and combinations of different FA species continue to challenge the broader understanding of the influence of FAs has on BrCa cell proliferation, migration, and invasion.

## 5 Conclusion

In conclusion, we revealed that extracellular FAs are used as both fuel and substrates for complex lipid synthesis such as TAG and ceramides in MCF‐7 and MDA‐MB‐231 cells. In addition, high extracellular lipid availability further enhanced FA flux in these cells, supported by an increase in ATGL protein levels. Furthermore, palmitate is preferentially incorporated into TAG for storage in the more aggressive MDA‐MB‐231 cells, whereas less aggressive cell line MCF‐7 shunts palmitate into FA oxidation, highlighting for the first time striking differences in FA metabolism between 2 commonly used BrCa cells. Indeed, inhibiting CPT1A activity blocked palmitate oxidation and increased palmitate toxicity in MCF‐7 cells, thus suggesting a potential therapeutic target for estrogen receptor α‐positive BrCa types which possess higher levels of CPT1A (Balaban *et al*., 2017). The increased complex lipid synthesis phenotype in MDA‐MB‐231 cells is enhanced in a lipid‐rich environment and prevents palmitate‐induced apoptosis, and therefore, this pathway represents a potential therapeutic target in these cells (Fig. 6G). The outcomes from these experiments may inform on the potential role that obesity‐associated dyslipidemia may play in influencing BrCa progression (De Angel *et al*., 2013; Hall *et al*., 2013; Liu *et al*., 2012), and therefore, these metabolic traits might be the basis for novel therapeutic targeting in BrCa, particularly in obese patients.

## Author contributions

SB, LSL, BV, AA, QG, and RFS performed experiments and analyzed data. DNS and TG provided intellectual input and edited the manuscript. AJH conceived the general ideas for this study, designed and performed experiments, analyzed data, and wrote the manuscript. All authors read and approved the final version of the manuscript.

## Supporting information


**Fig. S1.** Breast cancer cell response to palmitate supplementation.
