# Supplementary material for: Heterogeneity of fatty acid metabolism in breast cancer cells underlies differential sensitivity to palmitate‐induced apoptosis
Source: Mol Oncol. 2018 Aug 29;12(9):1623–38. doi: 10.1002/1878-0261.12368 (PMC6120225; doi:10.1002/1878-0261.12368)
Supplement: Supplementary file 1 — Fig. S1. Breast cancer cell response to palmitate supplementation. [file MOL2-12-1623-s001.docx]

**Heterogeneity of fatty acid metabolism in breast cancer cells underlies differential sensitivity to palmitate-induced apoptosis**

Seher Balaban ^1^, Lisa S. Lee ^1^, Bianca Varney ^1^, Atqiya Aishah ^1^, Quanqing Gao ^2^, Robert F. Shearer ^3^, Darren N. Saunders ^4^, Thomas Grewal ^2^, Andrew J. Hoy ^1 #^

^1^ Discipline of Physiology, School of Medical Sciences & Bosch Institute, Charles Perkins Centre, Faculty of Medicine and Health, The University of Sydney, NSW 2006, Australia.

^2^ School of Pharmacy, Faculty of Medicine and Health, The University of Sydney, Sydney, NSW 2006, Australia

^3^ Kinghorn Cancer Center, Garvan Institute of Medical Research, Darlinghurst, Australia

^4^ School of Medical Sciences, UNSW Australia, Sydney, NSW 2052 Australia.

**# Address for reprints and correspondence**:

Andrew J. Hoy, Ph.D.

Discipline of Physiology, Charles Perkins Centre, University of Sydney, NSW, Australia 2006

Phone: +61 2 9351 2514 Email: [andrew.hoy@sydney.edu.au](mailto:andrew.hoy@sydney.edu.au)


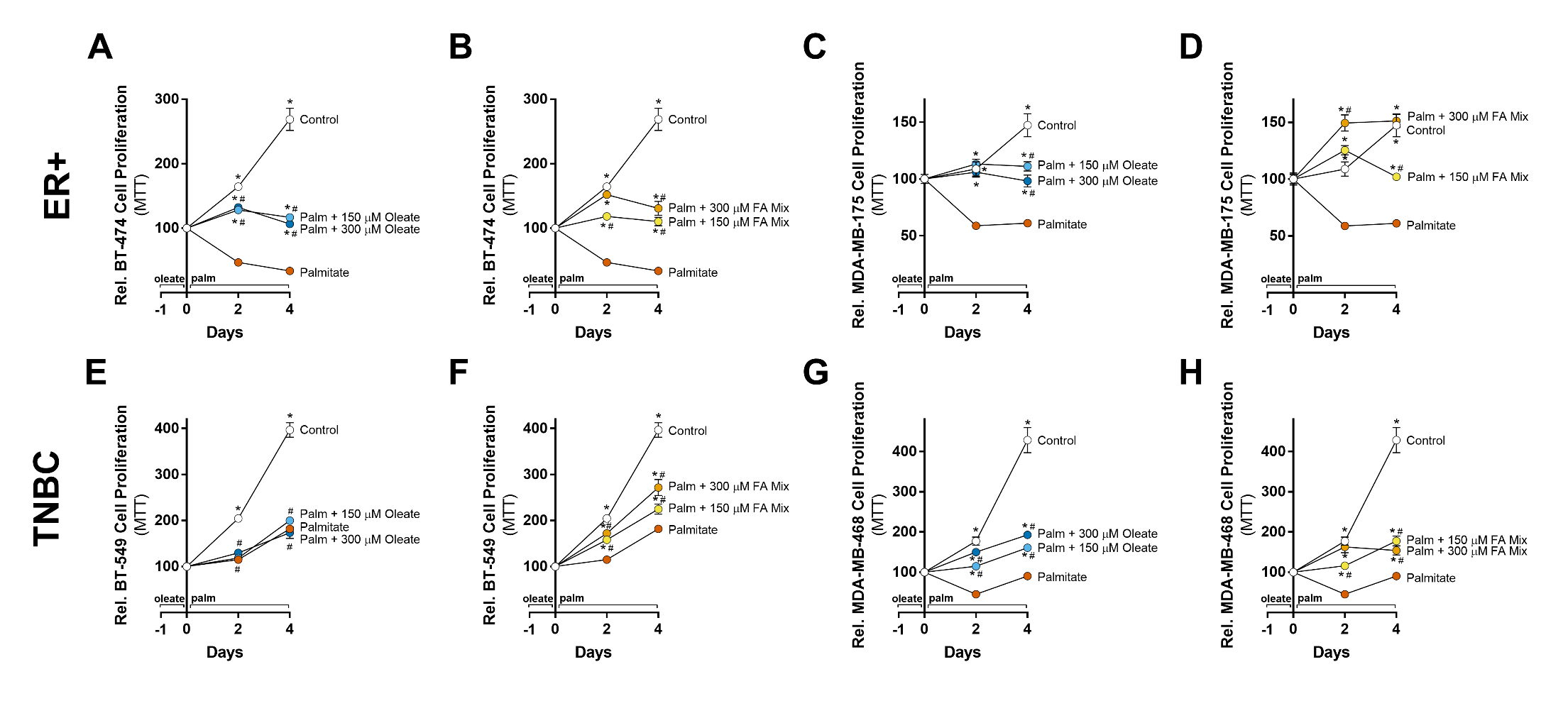
**SUPPORTING INFORMATION**

**Figure S1: Breast cancer cell response to palmitate supplementation.** MTT assays of **(A & B)** BT-474, **(C & D)** MDA-MB-175, **(E & F)** BT-549, and **(G & H)** MDA-MB-468 cells treated with 250 μM palmitate for 4 days with or without prior overnight incubation with **(A, C, E, G)** oleate or **(B, D, F, H)** 1:2:1 mixture of palmitate:oleate:linoleate (FA Mix). MTT results are presented as percentages of MTT absorbance at indicated time points relative to that at day 0 for each group (three independent experiments performed in quadruplicate). * *P* ≤ 0.05 vs. Palmitate; # *P* ≤ 0.05 vs. Control by two-way ANOVA followed by Tukey’s Multiple Comparisons test.
